# Unexpected presentation of ischemic colitis: a case report and review of the literature

**DOI:** 10.1093/jscr/rjad418

**Published:** 2023-07-12

**Authors:** Fatima Saoud Al-Mohannadi, Mohammed Al Obahi, Yaseen Al-Hashimy, Hijran Mahdi, Mohamed Badie Ahmed

**Affiliations:** Plastic Surgery Department, Hamad General Hospital, Hamad Medical Corporation, Doha, Qatar; Acute Care Surgery Department, Hamad General Hospital, Hamad Medical Corporation, Doha, Qatar; College of Medicine, QU Health, Qatar University, Doha, Qatar; Acute Care Surgery Department, Hamad General Hospital, Hamad Medical Corporation, Doha, Qatar; Acute Care Surgery Department, Hamad General Hospital, Hamad Medical Corporation, Doha, Qatar; Plastic Surgery Department, Hamad General Hospital, Hamad Medical Corporation, Doha, Qatar; College of Medicine, QU Health, Qatar University, Doha, Qatar

## Abstract

Ischemic colitis accounts for many cases of bowel infarction. Usually, it has various manifestations, such as vomiting, abdominal pain, hematochezia and many other symptoms. Risk factors might include age, medications, hypercoagulable state and chronic illnesses. However, it can still occur in healthy young patients. This might make it difficult for physicians to establish a correct diagnosis and generate the appropriate treatment plan for patients suffering from ischemic colitis. In this case we report a previously healthy 37-year-old female who had a sudden onset of lower abdominal pain associated with hematochezia. This patient was treated for upper respiratory tract infection by amoxicillin-clavulanate 2 weeks prior to her symptoms. The computed tomography abdomen findings along with the exploratory laparotomy confirmed the diagnosis of ischemic colitis. Ischemic colitis is a serious condition with high mortality and morbidity rate. Therefore, prompt investigation, and if indicated, surgical intervention should be calculated in sick patients complaining of abdominal pain and lower gastrointestinal bleeding.

## INTRODUCTION

Ischemic colitis (IC) is the most common condition of gastrointestinal (GI) ischemic insult. However, the incidence of IC is often underreported due to its vague presentation and similarity to other digestive tract diseases, such as infective colitis and inflammatory bowel disease [1]. Despite this underestimation, the incidence of IC has been found to have increased over the years from 6.1 cases/10 000 person-years in 1976–1980 to 22.9 cases/100 000 person-years in 2005–2009 [2]. Several predisposing factors for IC have been identified; however, they all share the same pathophysiology, which is colonic blood supply compromise. These factors include trauma, thrombosis, hypoperfusion states and mechanical obstruction [3, 4]. Another factor is pharmacologic agents, such as appetite suppressants, decongestants, cardiac glucosides, hormonal therapies, immunosuppressive agents, non-steroidal anti-inflammatory drugs (NSAIDs) and antibiotics [2].

In this case, we report a patient without any pre-existing medical conditions who experienced symptoms of IC (lower abdominal pain with hematochezia) 2 weeks post-treatment for upper respiratory tract infection (URTI) using amoxicillin-clavulanate (Augmentin) and NSAIDs.

## CASE PRESENTATION

A 37-year-old female patient presented to the Emergency Department (ED) with 2-day history of severe lower abdominal pain which radiated to bilateral flanks. The pain was associated with one episode of hematuria, multiple episodes of hematochezia, three episodes of vomiting and low-grade fever. The patient denied changes in bowel habits. She also complained of bilateral lower limb bruises. According to the patient, she had URTI and fever 2 weeks prior to her presentation, for which she was treated with 10 days of Augmentin and NSAIDs. She also denied family history of GI diseases. Surgical history is significant for cesarean section. On examination, she looked pale, hypotensive and not tachycardic. The abdomen was soft but distended with significant tenderness in her lower abdomen and digital rectal exam was positive for fresh blood and clots. Her laboratory results revealed anemia (hemoglobin 9.2) and thrombocytopenia (platelet 32). In ED, the patient developed two episodes of bleeding per rectum and therefore, resuscitation started, and she was transfused with 2 units of PRBC, 12 units of Platelets and 4 units of Fresh Frozen Plasma (FFP).

**Figure 1:**
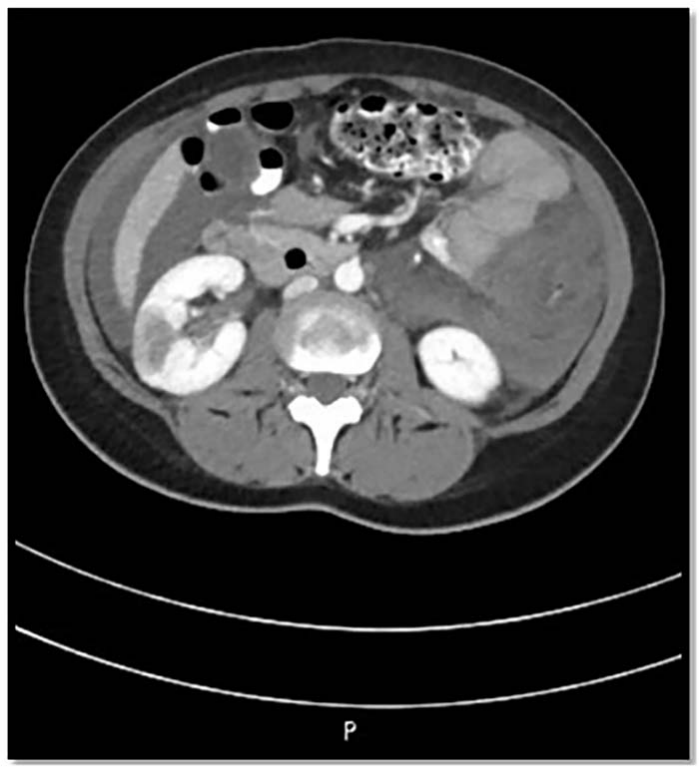
CT abdomen findings show significant thickening of the transverse colon with significant pericolic fat stranding; the right kidney shows wedge-shaped cortical hypodensities (could represent ischemic insult).

The patient was admitted to Surgical Intensive Care Unit (SICU) for hemodynamic monitoring and after a few hours of monitoring, she developed tachycardia, drop in hemoglobin and platelet count. Computed tomography (CT) angiogram abdomen was done that revealed hyperdense ascites, likely hemoperitoneum, nonenhancement of the sigmoid, descending, transverse and ascending colon along with the terminal ileum along with fat stranding suggestive of ischemia; however, there was no air in the mucosal wall or within the portal venous system. Also, Portal Vein (PV) and Superior Mesenteric Vein (SMV) thrombosis and bilateral kidneys wedge-shaped cortical hypodensities (mainly suggest ischemic insult) (Fig. 1).

**Figure 2:**
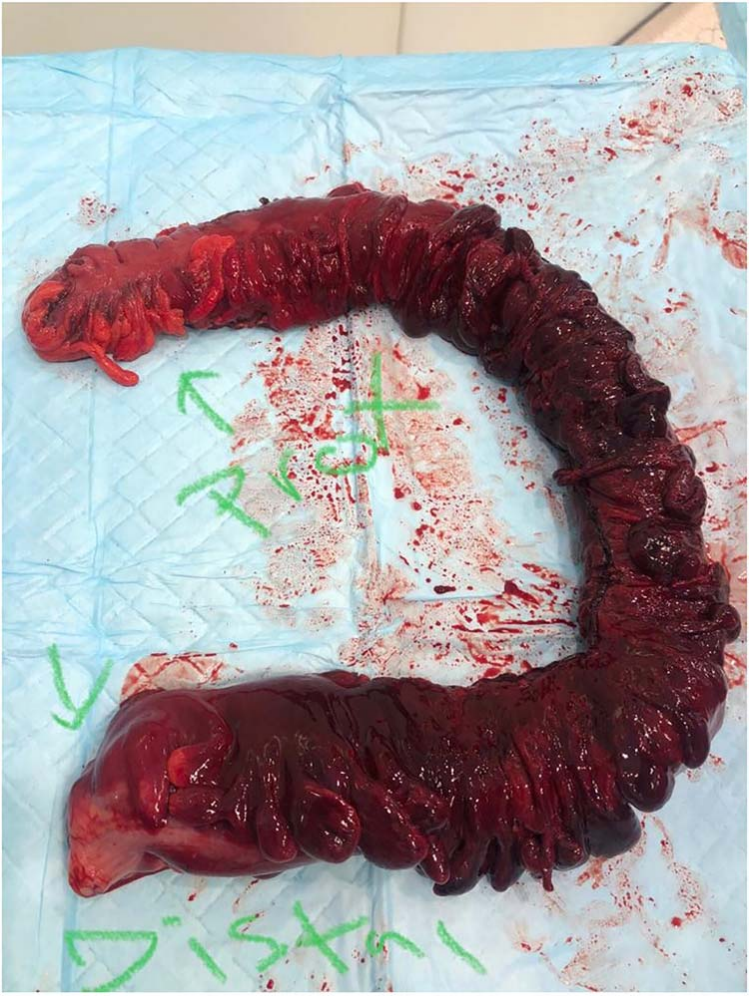
The full excised specimen from the exploratory laparotomy with left hemi and sigmoid colectomy, and transverse colostomy showing the proximal (transverse colon) and the distal (upper rectum) parts.

**Figure 3:**
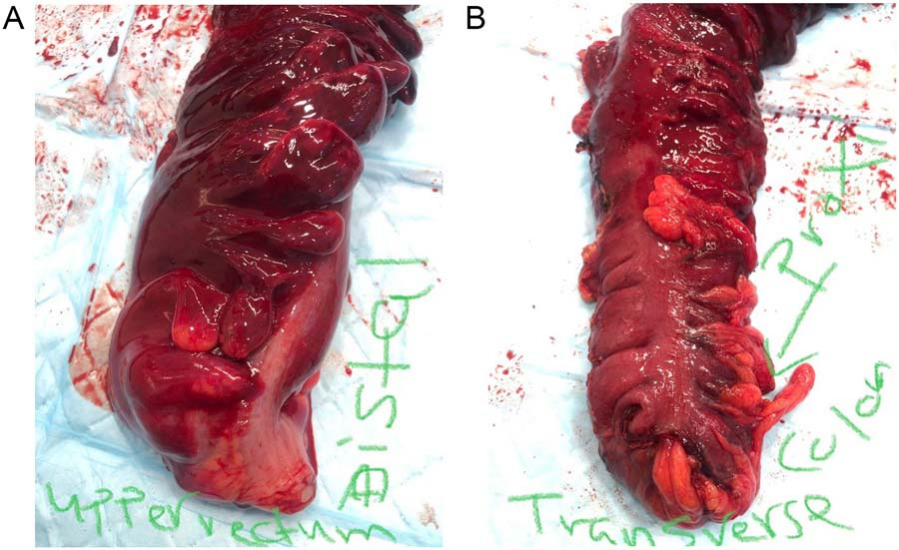
(left) Distal part of the excised specimen showing part of the upper rectum with healthy edge; (right) proximal part of the excised specimen showing part of the transverse colon with healthy edge.

Patient underwent exploratory laparotomy with left hemi and sigmoid colectomy, and transverse colostomy. Operative findings included gangrenous dilated colon from distal transverse colon to the proximal rectum with no perforation and around 2 l of hemoperitoneum and retroperitoneal hematoma (Figs 2 and 3). Specimen histopathology showed transmural necrosis and congestion and inflammation consistent with ischemia and bilateral viable margins.

Three days postoperatively, she developed dyspnea and oxygen desaturation. Imaging revealed Pulmonary Embolism; therefore, IV Heparin was administered. Then, US doppler of lower limbs was done that showed evidence of acute Deep Vein Thrombosis (DVT) on left side from proximal posterior tibial vein up to proximal superficial femoral vein. In addition, CT abdomen and pelvis was repeated but showed no resolution of PV/SMV thrombosis on anticoagulation and new renal vein thrombus. Thus, patient underwent suprarenal inferior vena cava filter insertion plus SMV, hepatic vein and PV AngioJet thrombolysis and thrombectomy. Autoimmune panel was negative for Lupus AC, ANA, ANCA, anti-B2GP, anti-cardiolipin, anti-dsDNA, anti-GBM and ASO titer. Also, IgG complements were normal.

Currently the patient is discharged, doing well on anticoagulation and will be assessed for underlying hidden malignancy as an outpatient.

## DISCUSSION

The colon can be affected, depending on the vascular insult severity. It can result in reversible transient IC, colonic gangrene or even fulminant universal. In addition, identifying the exact trigger of IC can be challenging, but it is often considered in 90% of elderly patients with chronic co-morbidities [1]. Conversely, our patient was a 37-year-old with no chronic illnesses. Other than co-morbidities, medications, including antibiotics, are considered a risk factor for IC and GI disturbances. Only one case of IC was reported in the literature following the immediate administration of Augmentin. It was found to be the result of an allergic reaction to the antibiotic which led to hypoperfusion and shock even though the patient has completed multiple courses of Augmentin in the past safely [5]. On the other hand, our patient took Augmentin 2 weeks prior to her presentation; therefore, an allergic reaction precipitating her IC is unlikely. Many medications have been linked to IC but were not consumed by our patient [6–8].

Moreover, hypercoagulability and thrombosis may also play a role in the development of IC in young patients regardless of the cause of the hypercoagulable state [9]. It usually presents as a sudden onset of abdominal pain and bleeding per rectum, which might be the result of mesenteric vein thrombosis, DVT or cerebrovascular insults [10]. Our patient was presented with abdominal pain and hematochezia, but she had negative thrombophilia workup and normal autoimmune panel which excludes hypercoagulability as a cause of her IC. On the contrary, imaging did show that she had both PV and mesenteric vein thrombosis. This led to difficulty in establishing the root cause of her condition. Other risk factors of IC that might have caused our patient’s symptoms are female sex and history of abdominal operations [11]. IC might also occur spontaneously in young and healthy patients with no clear explanation. In these cases, one could consider the anatomical variations of the bowel blood supply as some individuals might be more vulnerable to ischemia compared with others [1]. Overall, our patient did not have any condition that predisposed her to IC except consuming Augmentin and NSAIDs.

## CONCLUSION

In conclusion, it is crucial to consider the possibility of IC as a diagnosis in patients experiencing abdominal pain and hematochezia, even in the absence of co-morbidities or known risk factors. In this case we aim to highlight the importance of promptly identifying atypical manifestations of IC to prevent unnecessary surgical interventions and the resulting complications such as adhesions, strictures and potentially higher mortality rates.
